# Chronicity is associated with the glenohumeral synovitis in patients with a rotator cuff tear

**DOI:** 10.1002/jor.24941

**Published:** 2020-12-16

**Authors:** Du‐Han Kim, Ki‐Cheor Bae, Jung‐Hoon Choi, Sang‐Soo Na, Ilseon Hwang, Chul‐Hyun Cho

**Affiliations:** ^1^ Department of Orthopedic Surgery Keimyung University Dongsan Hospital, Keimyung University School of Medicine Daegu South Korea; ^2^ Department of Pathology Keimyung University Dongsan Hospital, Keimyung University School of Medicine Daegu South Korea

**Keywords:** glenohumeral joint, rotator cuff disease, subacromial space, synovitis

## Abstract

Synovitis of the glenohumeral joint (GHJ) and subacromial space (SAS) is one of the most common findings during arthroscopic rotator cuff repair (RCR). The purpose of this study is to determine clinical factors associated with the degree of synovitis in patients with a rotator cuff tear and whether macroscopic synovitis affects early clinical outcomes following arthroscopic RCR. Arthroscopic videos of 230 patients treated with arthroscopic RCR were randomly reviewed by two experienced shoulder surgeons. The synovitis scores of the GHJ using Davis's grading system and the SAS using Jo's grading system were rated with a consensus. Univariate and multivariate analyses were used to identify the associations between the synovitis scores and various parameters, including demographics, preoperative, and postoperative clinical outcomes. Univariate analyses revealed that age, side, body mass index, duration of symptoms, preoperative stiffness, diabetes, muscle atrophy, fatty infiltration, tear size, preoperative clinical scores, and preoperative range of motion were significantly associated with the GHJ synovitis score (all *p* < 0.05). Multivariate analyses revealed that the duration of symptoms, tear size, and diabetes was significantly associated with the GHJ synovitis score (*p* = 0.048, *p* = 0.025, *p* = 0.011, respectively). Longer duration of symptoms, larger tear size, and the presence of diabetes was independently associated with increased GHJ synovitis in patients with a rotator cuff tear. These results suggest that GHJ synovitis might be more involved in the pathogenesis for pain and tear progression of rotator cuff disease compared with SAS synovitis.

## 1 INTRODUCTION

Rotator cuff disease, the most common cause of shoulder pain and dysfunction, represents a spectrum of rotator cuff pathologies from tendinosis, partial‐thickness tear, full‐thickness tear, and rotator cuff tear arthropathy.^1^, ^2^, ^3^ The proposed etiology of rotator cuff disease includes degeneration, subacromial impingement, hypoxia, inflammation, and trauma.^3^, ^4^, ^5^ And its clinical manifestation and natural course vary widely among patients. While some patients experience rapid progression of tears with pain or functional disability, some have little or no progression of tears with minimal or no symptoms. However, the exact etiology and pathogenesis of rotator cuff disease remain unclear.

The rotator cuff is interposed between the glenohumeral joint (GHJ) and subacromial space (SAS) as a mover and stabilizer of the shoulder joint.^6^ It is generally recognized that rotator cuff disease involves not only the tendons but also tissues in the GHJ and SAS, including bursa, synovium, ligament, and joint fluid.^1^ Jo et al.^1^ suggested that rotator cuff disease, like osteoarthritis, is regarded and treated as a “pan‐joint disease” of the shoulder.

It is widely accepted that subacromial synovitis is a source of pain in rotator cuff disease.^6^, ^7^, ^8^, ^9^, ^10^, ^11^ The subacromial bursa is anatomically vulnerable to friction with the undersurface of the acromion during the range of motion (ROM) and synovitis occurs subsequently into the SAS.^12^ Several basic studies reported that overexpression of inflammatory cytokines, enzymes, and proteinases was observed in the subacromial bursa of patients with a rotator cuff tear.^6^, ^7^, ^8^, ^10^ These studies highlighted the important role that subacromial synovitis plays in the development of shoulder pain in patients with a rotator cuff tear and noted that its severity is associated with the pain intensity.^6^

A growing body of evidence exists for the role of GHJ synovitis in rotator cuff disease^3^, ^4^, ^12^, ^13^; however, it has not fully elucidated as of yet. Gotoh et al.^12^ noted increased expression of interleukin (IL)‐1β in synovial tissue in patients with a full‐thickness rotator cuff tear. Subsequently, several laboratory studies revealed that increased synovial inflammation and angiogenesis of the GHJ correlates with the tear size of the supraspinatus tendon and suggested that GHJ synovitis might be involved in the pathogenesis of rotator cuff tear.^3^, ^4^, ^13^

Synovial inflammation of the GHJ and SAS is one of the most common findings during arthroscopic surgery for a rotator cuff tear. Until now, most basic studies have characterized the biochemical and histologic findings of specimens including rotator cuff, synovium, joint fluid, or subacromial bursa.^4^, ^6^, ^7^, ^10^, ^12^, ^13^, ^14^, ^15^ Few studies have examined the macroscopic appearance of the synovial tissue of the GHJ and SAS.^1^, ^16^ Furthermore, no prior studies evaluated the potential association between macroscopic synovitis and various clinical factors in patients with a rotator cuff tear.

The primary aim of this study was to determine clinical factors associated with the degree of GHJ and SAS synovitis in patients with a rotator cuff tear. The secondary aim was to determine whether macroscopic synovitis affects early clinical outcomes following arthroscopic rotator cuff repair (RCR). This study was conducted to prove the hypothesis that the degree of macroscopic synovitis would correlate with clinical findings in patients with rotator cuff tear.

## 2 METHODS

This study was approved by the institutional review board of our hospital (IRB No:2020‐04‐026), and informed consent was obtained from all patients. Two‐hundred thirty patients who underwent arthroscopic RCR by a single surgeon at a single institution between October 2013 and February 2018 were included in this study. Inclusion criteria were as follows: (1) patients with arthroscopic RCR; (2) available medical records and arthroscopic findings; (3) available data for serial follow‐up periods including 3, 6, and 12 months after surgery. Exclusion criteria included: (1) a history of previous shoulder surgery or major trauma; (2) a history of inflammatory arthritis; (3) corticosteroid injection within 4 weeks before surgery; and (4) anti‐inflammatory medication within 2 weeks before surgery.

### 2.1 Macroscopic assessment for synovitis

With patients in the lateral decubitus position, a standard arthroscopic GHJ examination through the posterior and anterior portals to evaluate intra‐articular pathology was performed. Next, the arthroscope was placed in the SAS, and RCR was conducted. Using an arthroscopic shaver and radiofrequency device, arthroscopic debridement and ablation for the synovitis of the GHJ and SAS were performed as thoroughly as possible.

Arthroscopic videos of 230 patients treated with arthroscopic RCR were randomly presented to two shoulder surgeons for macroscopic assessment of synovitis. Before the independent assessment, the consensus for synovitis grading of the GHJ and SAS between two observers were generated through a detailed review of the studies reported by Davis et al.^16^ and Jo et al.^16^ with 30 samples of arthroscopic video. To evaluate intraobserver reliability, this same procedure was repeated 2 weeks after the first round of assessment.

**Figure 1:**
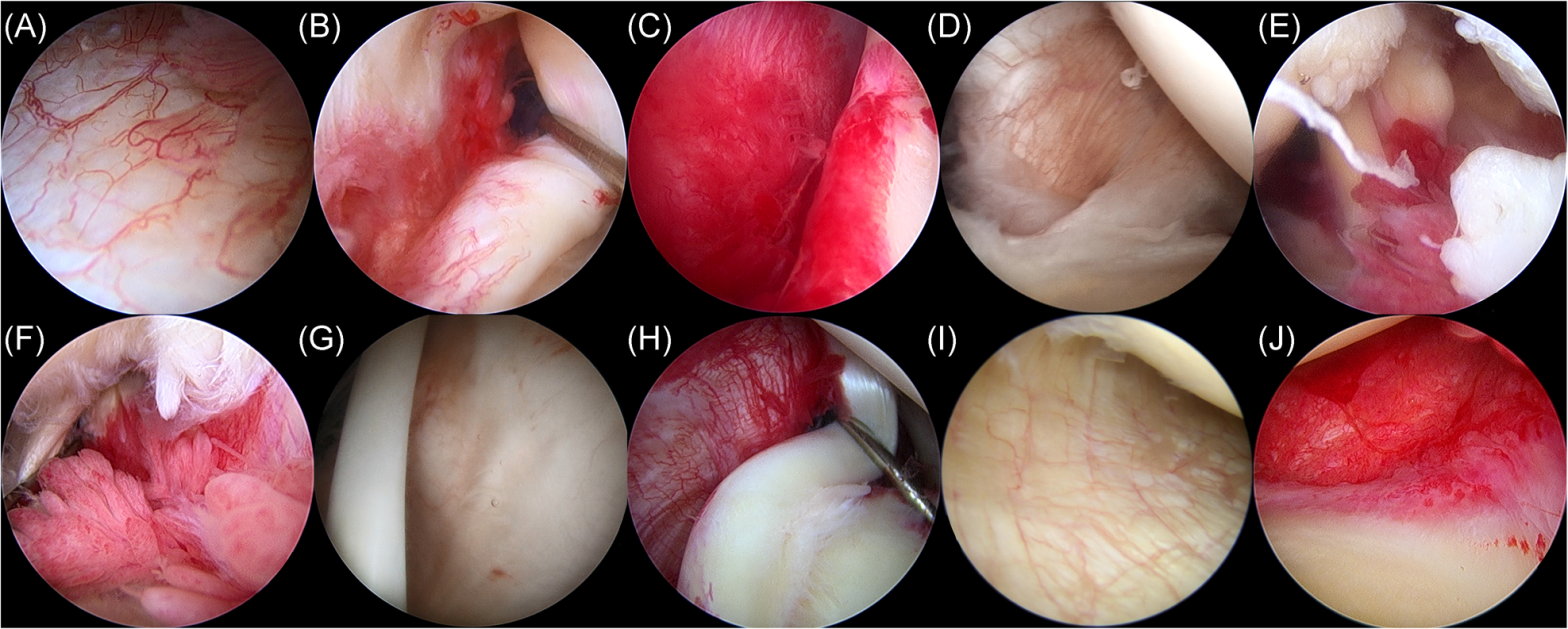
Macroscopic findings of the synovitis in the glenohumeral joint according to David's grading system. Color of the capsule; pale (A), pink (B), red (C). Villous projections; none (D), few (E), extensive (F). Capillaries in capsule; scattered (G), hypertrophied (H). Axillary recess; normal (I), contracted (J) [Color figure can be viewed at wileyonlinelibrary.com]

According to the grading system proposed by Davis et al.,^16^ GHJ synovitis was graded as follows: color of capsule (pale [0], pink [1], or red [2]); villous projections [none (0), few (1), or extensive (2)]; capillaries in capsule [scattered (0) or hypertrophied (1)]; and axillary recess [normal (0) or contracted (1)] (Figure 1). Total GHJ synovitis scores thus ranged from 0 to 6.

**Figure 2:**
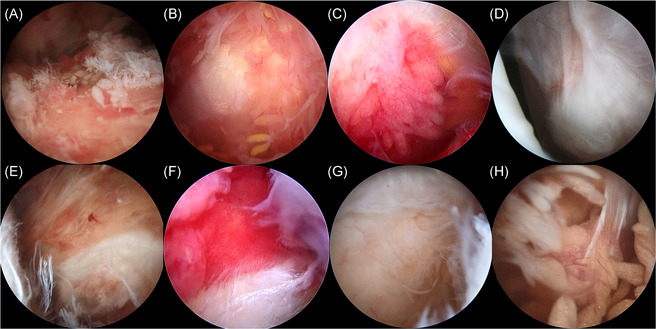
Macroscopic findings of the synovitis in the subacromial space according to Jo's grading system. Hypertrophy based on the size of the synovial villi; <2 mm (A), 2–5 mm (B), >5 mm (C). Hyperemia based on the redness of the villi; pale and transparent (D), slightly reddish (E), definitely red (F). Density assessed by the coverage of synovial villi; <1/3 (G), ≥ 1/3 (H) [Color figure can be viewed at wileyonlinelibrary.com]

According to the grading system proposed by Jo et al.,^1^ SAS synovitis was graded as follows: hypertrophy based on the size of the synovial villi [<2 mm (0), 2–5 mm (1), >5 mm (2)]; hyperemia based on the redness of the villi [pale and transparent (0), slightly reddish (1), definitely red (2)]; and density assessed by the coverage of synovial villi [>1/3 (0), ≥ 1/3 (1)] (Figure 2). Total SAS synovitis scores thus ranged from 0 to 5.

### 2.2 Clinical parameters

Available demographic and clinical parameters included age, sex, side, body mass index (BMI), occupation, duration of symptoms, history of trauma, preoperative stiffness, diabetes, muscle atrophy, fatty infiltration, tear size, preoperative and postoperative clinical scores, and ROMs. The occupation was divided into four categories for analysis (heavy work, light work, unemployed, others: refuse to reveal their occupation). We defined criteria for shoulder stiffness as follows: (1) passive forward flexion < 120˚°; (2) external rotation at side <30°; or (3) internal rotation at the back <third lumbar level according to the definition of Oh et al.^17^ Preoperative muscle atrophy was evaluated according to the Thomazeau classification.^18^ Fatty infiltration of muscle was evaluated according to the Goutallier classification.^19^ Tear size of the rotator cuff was classified according to DeOrio and Cofield.^20^, ^21^ Clinical evaluations included the visual analog scale (VAS) pain score (0 = no pain; 10 = unbearable pain), the American Shoulder and Elbow Surgeons' (ASES) score, and ROM assessment before surgery and at 3, 6, and 12 months after surgery. For statistical analysis of internal rotation, we converted values into contiguously numbered groups: 1–12 for T1–T12, 13–17 for L1–L5, 18 for the sacrum, and 19 for buttock.

### 2.3 Statistical methods

The SPSS statistical package (version 20.0; IBM) was used for data analysis. Intraobserver and interobserver reliability were assessed by calculating the κ correlation coefficient. To identify clinical parameters associated with the degree of synovitis, univariate analysis was conducted using the Pearson correlation test, Spearman correlation test, independent *t*‐test, and one‐way analysis of variance test. Significant associations observed in univariate analysis were included in the multivariate analysis. 95% confidence intervals were reported to provide the magnitude of the association. Statistical significance was accepted for *p* values of less than 0.05.

## 3 RESULTS

The mean age of patients was 60.4 ± 7.2 years (range, 42–76 years), and there were 132 women (57.4%) and 98 men (42.6%). The dominant side was involved in 172 patients (74.8%) and the nondominant side in the remaining 58 (25.2%). The mean BMI was 25.0 ± 3.0 kg/m^2^ (range, 17.0–32.9 kg/m^2^). The mean duration of symptoms was 30.0 ± 35.5 months (range, 1–168 months). Preoperative stiffness was observed in 40 patients (17.4%) and 32 (13.9%) had diabetes. Tear sizes were classified as partial‐thickness tear in 33 patients (14.3%), small tear in 33 (14.3%), medium tear in 74 (32.2%), large tear in 54 (23.5%), and massive tear in 36 (15.7%; Table 1).

**Table 1:** Univariate analysis between synovitis scores and clinical parameters

		*N* (%) or mean ± *SD*	GHJ synovitis score		SAS synovitis score	
			Mean ± *SD*	*p*	Mean ± *SD*	*p*
Age		60.4 ± 7.2		0.008*		0.395
Sex						
Man		98 (42.6%)	4.0 ± 1.5	0.400	1.9 ± 1.2	0.656
Woman		132 (57.4%)	3.8 ± 1.5		1.8 ± 1.2	
Side						
Dominant		172 (74.8%)	3.7 ± 1.5	0.011*	1.8 ± 1.2	0.360
Nondominant		58 (25.2%)	4.3 ± 1.2		2.0 ± 1.3	
Body mass index		25.0 ± 3.0		0.019*		0.105
Occupation						
Heavy work		98 (42.6%)	4.0 ± 1.4	0.425	1.8 ± 1.6	0.675
Light work		49 (21.3%)	4.0 ± 1.6		2.0 ± 1.2	
Unemployed×		81 (35.2%)	3.7 ± 1.5		1.8 ± 1.3	
Others		2 (0.9%)	3.0 ± 4.2		2.5 ± 0.7	
Sx duration		30.0 ± 35.5		0.027*		0.473
History of trauma						
No		191 (83.0%)	3.9 ± 1.4	0.471	1.8 ± 1.2	0.708
Yes (minor)		39 (17.0%)	3.7 ± 1.7		1.9 ± 1.1	
Preop stiffness						
No		190 (82.6%)	3.6 ± 1.5	0.006*	1.8 ± 1.2	0.334
Yes		40 (17.4%)	4.5 ± 1.4		2.0 ± 1.1	
Diabetes						
No		198 (86.1%)	3.7 ± 1.5	0.001*	1.8 ± 1.2	0.803
Yes		32 (13.9%)	4.7 ± 1.2		1.9 ± 1.2	
Muscle atrophy						
No ‐ mild		187 (81.3%)	3.7 ± 1.5	0.002*	1.8 ± 1.2	0.424
Moderate		39 (17.0%)	4.4 ± 1.4		1.9 ± 1.2	
Severe		4 (1.7%)	4.8 ± 1.0		2.5 ± 1.9	
Fatty infiltration						
No		1 (0.4%)	NA	0.022*	NA	0.839
Some		87 (37.8%)	3.7 ± 1.5		1.9 ± 1.1	
Evident		114 (49.6%)	3.9 ± 1.4		1.7 ± 1.2	
Fat = muscle		25 (10.9%)	4.3 ± 1.6		2.0 ± 1.4	
Fat > muscle		3 (1.3%)	5.0 ± 1.0		3.3 ± 1.2	
Tear size						
Partial		33 (14.3%)	3.2 ± 1.3	<0.001*	1.7 ± 1.0	0.641
Small		33 (14.3%)	3.5 ± 1.8		1.8 ± 1.3	
Medium		74 (32.2%)	4.0 ± 1.4		1.9 ± 1.3	
Large		54 (23.5%)	4.1 ± 1.4		2.0 ± 1.2	
Massive		36 (15.7%)	4.4 ± 1.3		1.7 ± 1.2	

Abbreviations: GHJ, glenohumeral joint; NA, not applicable; Preop, preoperative; SAS, subacromial space; *SD*, standard deviation; Sx, symptoms.

* Statistically significant.

The mean total GHJ synovitis score was 3.9 ± 1.5. For subitems, the mean score of color of capsule was 1.2 ± 0.6, villous projections 1.2 ± 0.6, capillaries in capsule 0.9 ± 0.3, and axillary recess 0.6 ± 0.5. The mean total SAS synovitis score was 1.9 ± 1.2. For subitems, the mean score of hypertrophy was 0.5 ± 0.6, hyperemia 1.1 ± 0.5, and density 0.2 ± 0.4. The intraobserver and interobserver reliability of GHJ synovitis grading system was color of the capsule (κ = 0.781 and 0.593), villous projections (κ = 0.615 and 0.694), capillaries in the capsule (κ = 0.694 and 0.530), and axillary recess (κ = 0.862 and 0.673; Tables 2 and 3). The intraobserver and interobserver reliability of the SAS synovitis grading system was hypertrophy (κ = 0.701 and 0.520), hyperemia (κ = 0.699 and 0.518), and density (κ = 0.773 and 0.666).

**Table 2:** Intraobserver reliability of the grading systems

	Observer 1	Observer 2	Mean κ‐value
GHJ synovitis grading			
Color of capsule	0.807	0.755	0.781
Villous projections	0.604	0.625	0.615
Capillaries in capsule	0.682	0.706	0.694
Axillary recess	0.848	0.876	0.862
SAS synovitis grading			
Hypertrophy	0.729	0.673	0.701
Hyperemia	0.701	0.697	0.699
Density	0.763	0.782	0.773

Abbreviations: GHJ, glenohumeral joint; SAS, subacromial space.

**Table 3:** Interobserver reliability of the grading systems

	First‐round	Second‐round	Mean κ‐value
GHJ synovitis grading			
Color of capsule	0.603	0.582	0.593
Villous projections	0.520	0.547	0.534
Capillaries in capsule	0.501	0.558	0.530
Axillary recess	0.689	0.657	0.673
SAS synovitis grading			
Hypertrophy	0.495	0.545	0.520
Hyperemia	0.512	0.523	0.518
Density	0.672	0.659	0.666

Abbreviations: GHJ, glenohumeral joint; SAS, subacromial space.

Univariate analyses revealed that age, side, BMI, duration of symptoms, preoperative stiffness, diabetes, muscle atrophy, fatty infiltration, and tear size were significantly associated with the GHJ synovitis score (all *p* < 0.05). Preoperative VAS pain score, ASES score, forward flexion, external rotation, and internal rotation were also significantly associated with the GHJ synovitis score (all *p* < 0.05). However, there were no associations between the SAS synovitis score and all parameters including demographics, preoperative, and postoperative clinical outcomes (all *p* > 0.05). There were no associations between the GHJ and SAS synovitis score and clinical outcomes at 3, 6, 12 months after surgery including VAS pain score, ASES score, and forward flexion, external rotation, and internal rotation (all *p* > 0.05; Table 4).

**Table 4:** Univariate analysis between synovitis scores and clinical scores

	Mean ± *SD*	GHJ synovitis grading		SAS synovitis grading	
		*r*	*p*	*r*	*p*
VAS pain score					
Preoperative	6.0 ± 2.2	0.133	0.044*	0.001	0.983
PO 3 months	3.6 ± 1.8	−0.029	0.665	−0.069	0.299
PO 6 months	2.5 ± 1.8	0.028	0.675	−0.066	0.319
PO 12 months	1.5 ± 1.5	−0.052	0.435	−0.029	0.664
ASES score					
Preoperative	44.3 ± 18.7	−0.251	<0.001*	0.013	0.847
PO 3 months	59.4 ± 14.4	0.026	0.693	0.035	0.601
PO 6 months	72.8 ± 14.8	−0.006	0.925	0.076	0.250
PO 12 months	84.9 ± 11.8	0.113	0.088	0.083	0.212
Forward flexion					
Preoperative	145.9° ± 31.4°	−0.229	<0.001*	−0.045	0.499
PO 3 months	143.1° ± 18.6°	0.028	0.667	−0.052	0.433
PO 6 months	158.3° ± 13.5°	0.007	0.921	0.002	0.973
PO 12 months	166.5° ± 8.3°	0.012	0.860	0.106	0.110
External rotation					
Preoperative	53.4° ± 23.4°	−0.180	0.006*	0.099	0.133
PO 3 months	51.9° ± 12.6°	−0.021	0.753	‐0.023	0.726
PO 6 months	64.0° ± 12.8°	−0.028	0.670	0.026	0.692
PO 12 months	72.2° ± 9.8°	−0.020	0.766	0.016	0.807
Internal Rotation					
Preoperative	12.4 ± 3.6	0.267	<0.001*	0.015	0.823
PO 3 months	13.4 ± 2.5	0.048	0.467	0.013	0.847
PO 6 months	10.6 ± 3.3	0.051	0.446	0.025	0.711
PO 12 months	8.2 ± 3.0	0.079	0.231	0.012	0.852

Abbreviations: ASES, American Shoulder and Elbow Surgeons; GHJ, glenohumeral joint; PO, postoperative; SAS, subacromial space; *SD*, standard deviation; VAS, visual analog scale.

* Statistically significant.

Multivariate analyses revealed that the duration of symptoms, diabetes, and tear size was significantly associated with the GHJ synovitis score (*p* = 0.048, *p* = 0.025, *p* = 0.011, respectively; Table 5).

**Table 5:** Multivariate analysis between synovitis scores and clinical parameters

	*t*	*p*	95% Confidence interval	
			Lower bound	Upper bound
Age	1.389	0.166	−0.007	0.043
Side	0.936	0.350	−0.214	0.600
Body mass index	0.531	0.596	−0.044	0.076
Duration of symptoms	1.990	0.048*	0.000	0.010
Preoperative stiffness	0.427	0.670	−0.461	0.715
Diabetes	2.258	0.025*	0.076	1.125
Muscle atrophy	1.833	0.068	−0.039	1.064
Fatty infiltration	−1.254	0.211	−0.617	0.137
Tear size	2.564	0.011*	0.052	0.394
Preoperative VAS pain score	−0.668	0.505	−0.245	0.121
Preoperative ASES score	−0.702	0.483	−0.038	0.018
Preoperative forward flexion	−0.721	0.472	−0.010	0.005
Preoperative external rotation	−0.912	0.363	−0.012	0.005
Preoperative internal rotation	1.482	0.140	−0.014	0.102

Abbreviations: ASES, American Shoulder and Elbow Surgeons; VAS, visual analog scale.

* Statistically significant.

## 4 DISCUSSION

The present study was conducted to identify clinical factors that may be associated with the degree of macroscopic synovitis in patients with a rotator cuff tear. The main findings were: (1) longer duration of symptoms, larger tear size, and the presence of diabetes were independently associated with increased GHJ synovitis; (2) SAS synovitis was not associated with any demographic and clinical parameters; (3) GHJ and SAS synovitis was not associated with postoperative clinical outcomes. These results suggest that GHJ synovitis might be more involved in the pathogenesis for pain and tear progression of rotator cuff disease compared with SAS synovitis.

Although significant biochemical and microscopic evidence of synovial inflammation of the GHJ and SAS in patients with a rotator cuff tear exists,^14^, ^15^ few studies have examined the macroscopic appearance of the synovial tissue of the GHJ and SAS. The absence of macroscopic studies may largely be due to the lack of a standardized grading system for synovitis as observed during arthroscopic surgery. Hence, Jo et al.^1^ proposed a macroscopic grading system for synovitis in the GHJ and SAS in patients with a rotator cuff tear and found that macroscopic findings were reliably correlated with microscopic findings. Subsequently, Davis et al.^16^ reported that the reliability of Jo's grading system may be low and proposed a new grading system for macroscopic synovitis of the GHJ with excellent reliability. Using these grading systems, it is possible to systematically score the degree of synovitis and to identify associations between macroscopic synovitis and various clinical factors. In the present study, considering the pros and cons of each system, we rated synovitis scores of the GHJ according to Davis's grading system and the SASaccording to Jo's grading system.

It is generally recognized that synovial inflammation of the SAS is associated with the pathophysiology of rotator cuff disease.^6^, ^7^, ^8^, ^9^, ^10^, ^11^ Gotoh et al.^6^ reported that IL‐1‐induced subacromial synovitis may play a role in shoulder pain. Blaine et al.^7^ reported that tumor necrotic factor (TNF)‐α, IL‐1α, IL‐1β, IL‐6, cyclooxygenase (COX)‐1, COX‐2, matrix metalloprotease (MMP)‐1, and MMP‐9 are overexpressed in the subacromial bursa in patients with a rotator cuff tear. SAS synovitis was associated with overexpression of proinflammatory cytokines, enzymes, and MMPs, which may have an important role in the pain mechanism and pathophysiology of rotator cuff tear.^7^, ^10^ Meanwhile, Gotoh et al.^12^ noted that overexpression of IL‐1β in the GHJ synovium in patients with a rotator cuff tear and emphasized the pivotal role of synovial inflammation in modulating rotator cuff degeneration. IL‐1β is well known to stimulate a cascade of catabolic responses by upregulating degradative enzymes including MMP‐1, MMP‐9, and MMP‐13.^3^ Indeed, recent studies reported an overexpression of MMP‐1 and MMP‐13 genes, involving cell‐mediated tendon degeneration, in the torn supraspinatus tendon and synovial fluid.^8^, ^14^ Shindle et al.^3^ reported that IL‐1β, IL‐6, COX‐2, MMP‐9, and vascular endothelial growth factor (VEGF) were overexpressed in the synovium of patients with a rotator cuff tear and suggested that chronic GHJ synovitis may be associated with rotator cuff tears. Abrams et al.^4^ reported increased synovial inflammation and angiogenesis, and upregulation of MMP‐3 in patients with a full‐thickness rotator cuff tear and found that expression of MMP‐3 correlates with the degree of synovitis.

Jo et al.^1^ found that the degree of macroscopic synovitis was significantly greater in the GHJ compared with the SAS. They reported this finding is unexpected and counter to conventional thinking since SAS synovitis was long considered a primary source of pain and pathophysiology of rotator cuff disease.^1^ In the present study, our results were consistent with those reported by Jo et al.^1^ Preoperative VAS pain score, ASES score, and all ROMs were significantly associated with the GHJ synovitis scores, not the SAS synovitis scores. Multivariate analyses revealed that longer duration of symptoms, larger tear size, and the presence of diabetes were independently associated with increased GHJ synovitis. However, the SAS synovitis was not associated with any tested parameters including demographics, preoperative clinical scores, and ROMs. Because there is little study to compare the expression of biochemical markers between SAS and GHJ, it is difficult to define the main contributing site associated with the pathophysiology of rotator cuff tear. However, very recently, biochemical studies have reported that overexpression of inflammatory cytokines, growth factors, enzymes, and MMPs in GHJ capsule and synovial fluid are associated with rotator cuff degeneration and tear progression as well as pain generation in patients with rotator cuff tear.^3^, ^4^, ^12^, ^14^ Based on the results from our study, we do not deny that synovial inflammation of the SAS is associated with the pathophysiology of rotator cuff disease. We think that the pathogenesis for pain in patients with rotator cuff tear may originate from the GHJ synovitis rather than the SAS synovitis.

Gotoh et al.^6^ observed that full‐thickness rotator cuff tears were associated with greater degrees of synovitis than partial‐thickness tears. Shindle et al.^3^ reported that increased synovial inflammation and tissue degeneration correlates with the tear size of the supraspinatus tendon. Tajana et al.^15^ reported that the total protein concentration of synovial fluid increased with the loss of integrity of the rotator cuff, reaching the highest levels in rotator cuff tear arthropathy. The absolute enzymatic activity of gelatinases was greater in full‐thickness tears compared with partial‐thickness tears.^15^ VEGF, a well‐known angiogenetic factor, plays an important role in the inflammation of synovial tissue.^11^, ^22^, ^23^ Yanagisawa et al.^11^ reported that VEGF expression was associated with vascularity, synovial proliferation, and pain in rotator cuff disease. VEGF expression was closely correlated with synovial proliferation and with neovascularization in Type II diabetics with rotator cuff disease.^22^ Our study also found that larger tear size and the presence of diabetes were independently correlated with increased GHJ synovitis. These findings suggest that the GHJ synovitis may be involved in degeneration and tear progression of the rotator cuff tendon. Further studies to characterize this relationship may help guide the development of effective treatments to reduce pain and prevent tear progression in patients with rotator cuff disease.

The potential effects of macroscopic synovitis on early clinical outcomes following arthroscopic RCR are not well understood. In the present study, arthroscopic debridement and ablation for the GHJ and SAS synovitis was performed as thoroughly as possible using an arthroscopic shaver and radiofrequency device. We found no associations between the GHJ and SAS synovitis score and clinical outcomes at 3, 6, 12 months after surgery including VAS pain score, ASES score, and all ROMs. However, this is a retrospective study without standard management guideline for synovitis. Further prospective randomized studies to determine whether macroscopic synovitis affects early clinical outcomes following arthroscopic RCR are warranted.

This study has several limitations. First, the synovitis score was only rated at the time of arthroscopic surgery, therefore, it was not possible to evaluate the serial effects of synovial inflammation on clinical symptoms and tear progression. Second, a potential correlation between macroscopic and microscopic evaluations was not characterized. Third, the degree of synovitis can vary with respect to location and grading of synovitis according to the location was not conducted. However, it is of note that this is the first study, to the best of our knowledge, to determine clinical factors associated with the severity of synovitis in a relatively large set of patients with a rotator cuff tear.

## 5 CONCLUSION

Longer duration of symptoms, larger tear size, and the presence of diabetes mellitus was independently associated with increased GHJ synovitis in patients with a rotator cuff tear. These results suggest that GHJ synovitis might be more involved in the pathogenesis for pain and tear progression of rotator cuff disease compared with SAS synovitis.

## AUTHOR CONTRIBUTIONS

Du‐Han Kim analyzed the data and reviewed the manuscript. Ki‐Cheor Bae reviewed the manuscript and supervision. Jung‐Hoon Choi gathered and analyzed the data and reviewed the manuscript. Sang‐Soo Na gathered and analyzed the data. Ilseon Hwang conceived the idea and analyzed the data. Chul‐Hyun Cho conceived the idea and wrote the manuscript.
